# Obesity is Associated with Reduced Plasticity of the Human Motor Cortex

**DOI:** 10.3390/brainsci10090579

**Published:** 2020-08-21

**Authors:** Sophia X. Sui, Michael C. Ridding, Brenton Hordacre

**Affiliations:** 1 Epi-Centre for Healthy Ageing, IMPACT Institute, School of Medicine, Deakin University, P.O. Box 281 (Barwon Health), Geelong VIC 3220, Australia; 2 Innovation, Implementation and Clinical Translation (IIMPACT) in Health, Allied Health and Human Performance, University of South Australia, Adelaide SA 5001, Australia; Michael.Ridding@unisa.edu.au (M.C.R.); Brenton.Hordacre@unisa.edu.au (B.H.)

**Keywords:** obesity, neuroplasticity, body mass index, transcranial magnetic stimulation, theta burst stimulation

## Abstract

Obesity is characterised by excessive body fat and is associated with several detrimental health conditions, including cardiovascular disease and diabetes. There is some evidence that people who are obese have structural and functional brain alterations and cognitive deficits. It may be that these neurophysiological and behavioural consequences are underpinned by altered plasticity. This study investigated the relationship between obesity and plasticity of the motor cortex in people who were considered obese (*n* = 14, nine males, aged 35.4 ± 14.3 years) or healthy weight (*n* = 16, seven males, aged 26.3 ± 8.5 years). A brain stimulation protocol known as continuous theta burst transcranial magnetic stimulation was applied to the motor cortex to induce a brief suppression of cortical excitability. The suppression of cortical excitability was quantified using single-pulse transcranial magnetic stimulation to record and measure the amplitude of the motor evoked potential in a peripheral hand muscle. Therefore, the magnitude of suppression of the motor evoked potential by continuous theta burst stimulation was used as a measure of the capacity for plasticity of the motor cortex. Our results demonstrate that the healthy-weight group had a significant suppression of cortical excitability following continuous theta burst stimulation (cTBS), but there was no change in excitability for the obese group. Comparing the response to cTBS between groups demonstrated that there was an impaired plasticity response for the obese group when compared to the healthy-weight group. This might suggest that the capacity for plasticity is reduced in people who are obese. Given the importance of plasticity for human behaviour, our results add further emphasis to the potentially detrimental health effects of obesity.

## 1. Introduction

Obesity, characterised by excessive body fat, affects a high proportion of the global population and is associated with decreased life expectancy [1]. Obesity has been linked to increased likelihood of cardiovascular disease, metabolic disorders, and dementia [2,3,4]. Given the growing and significant number of people who are obese and the associated medical complications, obesity imposes a significant financial burden on society and is therefore a major health concern.

In animal studies, obesity induced by genetic alteration or dietary methods has been shown to create significant functional or structural changes to specific areas of the brain, such as the hippocampus [5,6] and the hypothalamus [7]. Animal studies suggest that obesity is associated with increased susceptibility to neurodegeneration [5], reduced brain weight and cortical brain volume, and delayed maturation of neurons and glial cells [8]. Furthermore, adipose tissue is known as a source of proinflammatory cytokines, and there is some evidence that inflammation induced by obesity can lead to synaptic dysfunction [9].

In humans, there is some indication from neuroimaging studies that obesity may increase the odds of developing age-related white matter changes [10]. Furthermore, there is additional evidence that shows that brain-derived neurotropic factor (BDNF) levels or signaling are reduced in obesity [11]. This is important because BDNF appears to play a critical role in synaptic plasticity as it regulates neural circuit development and function [12]. While this indirect evidence appears to suggest that plasticity may be impaired in obesity, studies are required to verify this hypothesis. It may be particularly important to address this question as plasticity is an important aspect of human brain function that supports learning and memory. Synaptic plasticity is one mechanism of plasticity and refers to the ability to change the strength of synaptic connections in neuronal networks. Two forms of activity-dependent changes in synaptic strength—namely, long-term potentiation, which is an increase in synaptic strength, and long-term depression, which is a decrease in synaptic strength—have been identified as critical mechanisms underpinning learning and memory [13]. Non-invasive brain stimulation provides an opportunity to investigate plasticity in humans by providing a brief burst of repeated stimuli to induce neural changes that resemble synaptic plasticity. Theta burst stimulation is one such technique, with previous pharmacological studies providing some indication that the physiological response to stimulation is dependent on N-Methyl-d-aspartate (NMDA) receptors and resembles an early form of synaptic plasticity [14]. Thus, the aim of this study was to examine whether obesity is associated with an impaired response to theta burst stimulation in humans. If obesity is associated with a reduced response to theta burst stimulation, it would provide the first physiological evidence of reduced plasticity and further emphasise the negative health effects of obesity. It was hypothesised that people who are obese will have a reduced response to theta burst stimulation compared to people who are of a healthy weight.

## 2. Methods

### 2.1. Study Design and Participants

For this study, we purposively recruited people who were obese and an age- and gender-matched healthy-weight control group. Participants were recruited from the Comprehensive Metabolic Care Centre at Royal Adelaide Hospital or the wider community or through advertisements placed across the University of Adelaide. The inclusion criteria were that all participants must be aged between 18 to 60 years of age, not have a diagnosed neurological condition or musculoskeletal impairment of the upper limb, not currently take neuroactive medication, and have no contraindications for transcranial magnetic stimulation (TMS), such as metallic implants, history of seizures, or an implanted permanent pacemaker [15]. Although previous data were not available to inform a power calculation, we determined the sample size required based on a 20% reduction in plasticity in people who are obese compared to healthy adults (population variance 0.15). To achieve 80% power and significance of *p* < 0.05, a total sample of 30 participants (15 per group) was required. Ethical approval was provided by the Royal Adelaide Hospital human research ethics committee and the University of Adelaide human research ethics committee (HREC/15/RAH/26). The study complied with the Declaration of Helsinki, and all participants provided written informed consent.

### 2.2. Experimental Procedures

Participants attended the Neuromotor Plasticity and Development laboratory of the University of Adelaide for neurophysiological experiments (i.e., TMS testing). The sessions were scheduled after 12 p.m. to minimise time-of-day effects on plasticity responses [16]. During the neurophysiological experiment, participants were seated comfortably in a large chair with their right arms rested in a relaxed position.

### 2.3. Participant Characteristics

A standard questionnaire was used to collect demographic data from each participant. At the beginning of the experiment, age, gender, and handedness (self-reported) were documented. Participants’ weight, height, and percentage of body fat were measured using bio-impedance scales and a body composition analyser (Tanita, Kewdale, Australia). The body mass index (BMI: weight/height^2^) and waist-to-hip ratio of each participant were calculated. Participants were grouped according to their BMI: healthy weight (BMI = 18.5–24.99) or obese (BMI ≥ 30) [17]. Data were also recorded using the International Physical Activity Questionnaire (IPAQ) [18] and Pittsburgh Sleep Quality Index (PSQI) [19] for measuring recent levels of physical activity and sleep quality, respectively. The IPAQ requires participants to self-report physical activity over the previous 7 days in the domains of walking, moderate intensity, and vigorous intensity. The total score is computed based on the summation of the duration (in minutes) and frequency (in days) of all three activity types. The volume of activity is determined by weighting the three activity types (walking, moderate intensity, and vigorous intensity) by the energy requirements defined in multiples of the resting metabolic rate (METs). This calculation provides a total score in MET-minutes, with higher values indicative of greater physical activity. The PSQI is a self-reported questionnaire that assesses sleep quality over a one-month period. It includes 19 items grouped into seven domains, with each domain scored in the range 0–3. The total PSQI score can range from 0 to 21, with lower scores suggestive of better sleep quality.

### 2.4. Electromyography

Surface electromyographic (EMG) activity was recorded by attaching standard silver chloride surface electrodes (Ambu, Ballerup, Denmark) to the skin overlying the first dorsal interosseous (FDI) muscles of the right hand in a belly-tendon montage. The skin overlying the FDI was cleaned with alcohol and lightly abraded with NuPrep paste prior to fixing the EMG electrodes. The EMG signals were sampled at 5 kHz, amplified with a gain of 1000, filtered between 20 and 1000 Hz (Cambridge Electrical Design 1401 and 1902, Cambridge, UK), and stored on a computer for further analysis offline (version 5.01, Signal software, Cambridge Electronic Design, Cambridge, UK).

### 2.5. Transcranial Magnetic Stimulation

A Magstim 200 magnetic stimulator and a figure-of-eight coil (Magstim, Dyfed, UK) were used to apply single monophasic TMS pulses to the left motor cortex. The coil was held tangentially to the scalp with the handle pointing posterolateral at a 45-degree angle to the sagittal plane to induce a posterior-to-anterior current flow across the hand motor cortex. The optimal position for evoking motor evoked potentials (MEPs) in the relaxed FDI was identified by systematically moving the coil in small increments anterior–posterior, medial–lateral, and rotating the handle. Once the optimal position was identified, it was marked on the scalp with a felt tip pen for reference during the following experimental process to ensure consistent coil placement.

Baseline measures of corticospinal excitability were established and included the resting motor threshold (RMT), which was defined as the lowest stimulus intensity (percentage of maximum stimulator output, MSO%) that evokes MEPs of at least 50 µV from the FDI at rest in at least 5 of 10 consecutive trials. The stimulus intensity sufficient to evoke an MEP of 1 mV peak-to-peak amplitude (SI_1mV_) was also established. To assess corticospinal excitability at baseline, two blocks of 15 MEPs were recorded using intensity SI_1mV_. A short rest (~1 min) occurred between the two blocks, and the mean peak-to-peak MEP amplitude was determined. The latency of MEP onset of baseline MEPs was also determined as a method to investigate nerve entrapment syndromes, such as carpal tunnel syndrome, which can be more common in people who are obese [20]. Similar to our previous work, the MEP onset latency was determined automatically using a custom-made script to avoid assessor bias (Signal v4.09, Cambridge Electronic Design, Cambridge, UK) [21]. The MEP onset latency was defined as the time point where the rectified EMG signals increase above a threshold of 0.01 mV within a window of 15–40 ms after the TMS pulse. Following theta burst stimulation, corticospinal excitability was determined using the same stimulus intensity (SI_1mV_) used in baseline measurements. Four blocks of 15 MEPs were recorded at 0, 5, 15, and 30 min after theta burst stimulation.

### 2.6. Theta Burst Stimulation

Spaced continuous theta burst stimulation (cTBS) was used to induce plasticity within the left motor cortex. cTBS produces a transient reduction in corticospinal excitability that is thought to reflect an early form of long-term depression synaptic plasticity [14]. The application of a spaced cTBS protocol consists of two cTBS protocols applied 10 min apart and has been shown to induce a longer-lasting and more robust plasticity response [22,23,24]. cTBS was applied to the left motor cortex using an air-cooled figure-of-eight coil connected to a Magstim Super Rapid stimulator (Magstim, Whitland, Dyfed, UK). Each cTBS protocol consisted of three stimuli delivered at 50 Hz presented every 200 ms during a period of 40 s, delivered in a continuous manner (a total of 600 stimuli) [25]. Similar to in previous studies, the intensity of stimulation was set up to 70% of RMT-biphasic, which was determined using the same Magstim Super Rapid and air-cooled coil as used for the delivery of cTBS [22]. Participants remained relaxed during all neurophysiological experimental procedures, and the EMG was visually monitored to ensure that muscles were at rest.

### 2.7. Data Analysis and Statistics

The IBM SPSS Statistics package (V26, Chicago, IL, USA) was used to perform all statistical testing. The data were checked for normality using the Shapiro–Wilk test. Non-normalised data were analysed using non-parametric statistics. Participants were categorised as having a healthy weight or being obese based on BMI. Demographics and neurophysiological measures were compared between people of healthy weight and people who were obese using either independent t-tests (waist–hip ratio, RMT, SI_1mV_, baseline MEP amplitude, MEP onset latency), Mann–Whitney U tests (age, BMI, body fat%), or Fisher’s exact tests (gender, self-reported handedness). For the healthy-weight and obese groups, individual cTBS response was determined as the mean post-cTBS MEP amplitude at each time point (0, 5, 15, and 30 min post-cTBS) relative to the baseline MEP amplitude. A 2 GROUP (healthy, obese) × 4 TIME POINT (0, 5, 15, and 30 min post-cTBS) repeated-measures ANOVA was performed to investigate cTBS responses. Where appropriate, Bonferroni corrected t-tests were performed for post hoc analyses. The level of significance was set at *p* ≤ 0.05.

## 3. Results

### 3.1. Participant Characteristics

The participant demographics, characteristics, and baseline neurophysiological data are provided in Table 1. Participants in the obese group had significantly greater BMI, higher body fat percentage, and larger waist–hip ratios when compared to those in the healthy group (all *p* < 0.010, Table 1). There were no significant differences between the groups in terms of self-reported handedness, gender, or age (all *p* > 0.070, Table 1). There were no significant differences between groups for RMT, SI_1mV_ (% MSO), baseline MEP amplitude, or MEP onset latencies (all *p* > 0.263, Table 1).

### 3.2. cTBS Responses

**Figure 1:**
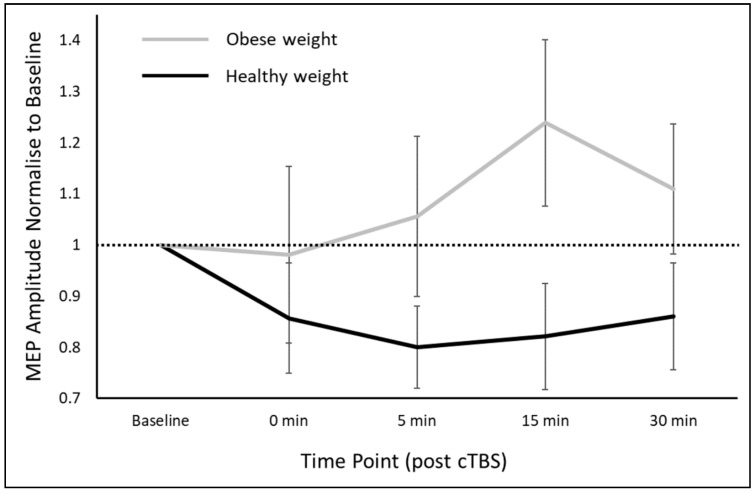
Comparison of the cTBS response between healthy-weight adults and people who are obese. There was a GROUP effect with healthy-weight participants demonstrating the expected suppression of MEP amplitudes following cTBS. Data are expressed as group mean ± SD. cTBS, continuous theta burst stimulation; MEP, motor evoked potential.

The repeated-measures ANOVA revealed a significant main effect of GROUP (F_(1,28)_ = 3.50, *p* = 0.036), but no effect of TIME POINT (*p* = 0.601) or GROUP × TIME POINT (*p* = 0.477) interaction. Post hoc analysis revealed a significantly greater cTBS response for participants in the healthy-weight group compared to the obese group (cTBS response; healthy-weight group 0.83 ± 0.32, obese group 1.09 ± 0.43; t_(28)_ = 1.85, Cohen’s d = 0.69). On further investigation, people in the healthy-weight group had a reduction in MEP amplitudes from baseline (1.01 ± 0.21 mV) to post-cTBS (0.83 ± 0.34 mV) that reached significance (t_(15)_ = 2.20, *p* = 0.044, Bonferroni corrected). However, people in the obese group did not appear to have a reduction in cortical excitability, with the difference between baseline (0.98 ± 0.32 mV) and post-cTBS (1.05 ± 0.51 mV) MEP amplitudes not reaching significance (t_(13)_ = −0.68, *p* = 1.00, Bonferroni corrected). Thus, it appeared that the participants in the healthy-weight group had a significant suppression in MEP amplitude following cTBS, but this was not observed for those in the obese group. As such, those in the healthy-weight group had a significantly stronger suppressive response to cTBS when compared to those in the obese group. The cTBS response for the healthy-weight and obese groups is shown in Figure 1.

## 4. Discussion

To our knowledge, this is the first study to investigate whether individuals who are obese have reduced capacity for plasticity in the primary motor cortex. Our results provide evidence that obesity is associated with impaired synaptic plasticity as we did not observe a change in cortical excitability following cTBS in people who are obese, but we did see a significant suppression of the MEP for people who are of healthy weight. Obesity appears to have a medium size effect on cTBS response (Cohen’s d = 0.69), suggesting that future studies should consider this an important variable that might influence neuromodulation with brain stimulation. This reduced plasticity in obese individuals may be due to changes in structural and functional brain characteristics as a result of the underlying pathophysiological consequences of obesity. These results provide further evidence to highlight the harmful effects of obesity on human health and provide further insight to understand patient-level characteristics that appear to influence the response to neuromodulatory brain stimulation protocols such as theta burst stimulation [21,26,27,28,29].

The physiological response to cTBS provides a reasonable surrogate to assess plasticity in humans. There is good evidence that spaced cTBS can produce a robust suppression of corticospinal excitability for a brief period following stimulation [22,23,24]. The aftereffects of cTBS can be blocked by the administration of NMDA receptor antagonists, suggesting that the suppression of corticospinal excitability is likely due to long-term-depression-like mechanisms that might represent an early phase of synaptic plasticity [14]. Furthermore, there is some evidence indicating that the aftereffects of cTBS are cortical in origin. Corticospinal volleys evoked by TMS were recorded with cervical epidural electrodes, and it was observed that cTBS reduced the amplitude of early I-waves [30]. This might suggest that intrinsic neural circuits of the motor cortex are responsible for the suppression of corticospinal excitability. Therefore, the magnitude of response to cTBS provides some indication of the ease of changing synaptic efficiency in the motor cortex and can therefore be considered a measure of plastic capacity. Since the suppression of corticospinal excitability by spaced cTBS is relatively robust [22,24], it is particularly noteworthy that people who are obese had a poor response. This might suggest that the capacity for synaptic plasticity is reduced in people who are obese.

Our findings suggesting that obesity is associated with impaired plasticity are well supported by the previous literature. For example, in humans, imaging studies have reported that obesity is associated with reduced cortical thickness [31]. This could lead to neuronal reduction, likely reducing the capacity for synaptic plasticity. Along similar lines, there is evidence that obesity can increase susceptibility to developing age-related white matter changes [10]. Furthermore, obesity has been linked with poorer cognition, impaired memory, and deficits in learning [32,33,34,35]. Our findings of reduced capacity for plasticity could be viewed as a link between the reported structural brain changes and both cognitive and behavioral abnormalities in people who are obese.

What may be the mechanism by which obesity impairs capacity for plasticity? A reduction in BDNF level or expression may be one candidate mechanism to explain our findings. Obesity is associated with reduced levels of BDNF or reduced BDNF signaling, which are associated with deficits in neuronal and behavioral plasticity [11]. As evidence of this, acute delivery of BDNF has been shown to affect synaptic transmission and plasticity [36,37]. This may be because BDNF potentiates excitatory synapses by increasing presynaptic glutamate release and augmenting postsynaptic NMDA receptor activity [38,39,40,41,42,43]. Furthermore, acute BDNF reduces inhibitory synaptic activity, increasing the potential for synaptic plasticity [44,45]. Another mechanism linking obesity and impaired synaptic plasticity is inflammation. Systemic and central inflammation is associated with obesity and has been linked with impaired synaptic plasticity and cognitive decline [11]. Pro-inflammatory cytokines (e.g., interleukin 1) derived from adipose tissue suppress neuronal function in the brain, thus impairing synaptic plasticity and cognitive function [9]. For example, a study found that surgical fat transplantation in rats resulted in systematic and central inflammation, which adversely affected synaptic plasticity and cognitive function, suggesting that inflammation mediates the impairment of cognitive and synaptic plasticity [9]. The roles of both BDNF and inflammation are worthy of investigation as mechanisms mediating the observed association between impaired plasticity and obesity.

There are some important implications from our findings. While several studies have provided evidence that obesity is detrimental for several aspects of human health [2,3,4], our findings further emphasise these negative health consequences by highlighting the association with impaired plasticity. This finding might suggest that weight reduction is particularly important in various clinical patient groups where learning is fundamental for recovery, such as after stroke or in mild cognitive impairment. However, we acknowledge that further studies are required to demonstrate a causal relationship between obesity and impaired synaptic plasticity. Such studies quantifying plasticity across a weight loss intervention would have significant benefits in providing insight into brain health. Furthermore, as this study only examined an inhibitory (LTD) form of plasticity, facilitatory plasticity (long-term potentiation; LTP) should be explored in individuals who are obese; additionally, cortical excitability and intracortical circuits should also be explored to gain further mechanistic insight that might help to further explain the current findings.

### Limitations

There are several limitations to this study that should be acknowledged. First, this was a cross-sectional study that recruited a relatively small sample. Future studies should seek to conduct larger investigations that investigate plasticity with a weight loss intervention. Such work would provide greater insight to understand the causal relationship between obesity and plasticity. Furthermore, while physical activity levels appeared to be similar between the groups and are therefore unlikely to have contributed substantially to impaired plasticity in the obese group for this study, we acknowledge that there may be unaccounted-for factors that could have contributed to the reduced plasticity response in the people who are obese. Such factors might include the presence of type 2 diabetes, which is common in obesity, leads to reduced secretion of insulin and hyperglycaemia, and may influence plasticity [46]. Future studies should screen for the presence of type 2 diabetes to help interpret the current results. Furthermore, obstructive sleep apnoea can be more common in people who are obese and may lead to brain changes [46] but was not quantified in this study. However, we note that the PSQI appeared to indicate that sleep quality did not differ between the groups. Finally, we acknowledge that our measure of plasticity is specific to the motor cortex and a long-term-depression-like form of synaptic plasticity. It may be that plasticity for other brain regions or different mechanisms of plasticity could behave differently in people who are obese. Finally, we also acknowledge that it may be that our results do not represent impaired synaptic plasticity in people who are obese, but rather, it could be observed that there is a role of homeostatic plasticity in governing the cTBS responses we have observed [47,48,49]. However, in our study, the baseline cortical excitability was similar between the groups, and there was no active task to induce neural activity during the procedures. While this could be a mechanism, further investigation is required to understand the role of homeostatic plasticity in obesity.

## 5. Conclusions

In conclusion, this is the first study to investigate the association between obesity and plasticity. Preliminary analysis showed that the capacity for synaptic plasticity is reduced in people who are obese when compared to those of healthy weight. Given the significant and increasing prevalence of obesity, this finding of reduced capacity for plasticity has significant implications for brain health for many people.

## Figures and Tables

**Table 1:** Participant demographics, characteristics, and baseline neurophysiological data.

	Healthy	Obese	Statistics
**Demographic data**	*n* = 16	*n* = 14	
Handedness (*n*, right/left)	(14:2)	(11:3)	*p* = 0.642
Gender (*n*, male/female)	(7:9)	(9:5)	*p* = 0.299
Age (years, mean (SD))	26.3 (8.5)	35.4 (14.3)	U = 68.5, *p* = 0.070
IPAQ total (MET mins, mean (SD))	4487.6 (5339.1)	5381.7 (9229.5)	U = 106.0, *p* = 0.822
PSQI (mean (SD))	4.25 (3.17)	6.14 (3.72)	U = 73.5, *p* = 0.110
BMI (kg/m^2^, range)	22.0 (20.0–24.9)	43.7 (30.0–68.0)	U = 0.0, ***p* < 0.001**
Percentage of body fat (%, mean (SD))	19.7 (6.4)	36.1 (15.3)	U = 32.0, ***p* = 0.010**
Waist–hip ratio mean (SD)	0.81 (0.06)	0.95 (0.06)	t_(28)_ = 6.16, ***p* < 0.001**
**Baseline excitability data**			
RMT (% MSO, mean (SD)	41.4 (7.0)	43.9 (5.0)	t_(28)_ = 1.11, *p* = 0.279
SI_1mV_ (% MSO, mean (SD))	57.4 (14.6)	62.6 (9.3)	t_(28)_ = 1.14, *p* = 0.263
Baseline MEP amplitude (mV, mean (SD))	1.01 (0.21)	0.98 (0.32)	t_(28)_ = 0.32, *p* = 0.750
MEP onset latency (ms, mean (SD))	24.16 (2.52)	24.25 (1.77)	t_(28)_ = 0.11, *p* = 0.916

Statistically significant differences are shown with bold *p*-values. BMI, body mass index; IPAQ, International Physical Activity Questionnaire; PSQI, Pittsburgh Sleep Quality Index; ms, milliseconds; MSO, maximum stimulator output; RMT, resting motor threshold; SI_1mV_, Stimulus intensity required for motor evoked potential (MEP) of 1 mV peak-to-peak amplitude.
